# Emerging Roles of Exosomes in Cancer for Possible Clinical Use

**DOI:** 10.3390/cancers14194603

**Published:** 2022-09-22

**Authors:** Hedwig Sutterlüty, Klaus Holzmann

**Affiliations:** Center for Cancer Research, Comprehensive Cancer Center, Medical University Vienna, Borschkegasse 8a, 1090 Vienna, Austria

Exosomes are membrane-structured extracellular vesicles (EVs) with nano-scale size that are released from different cell types [1]. The contents of exosomes reflect the donor cells with their microenvironment. Exosomes play a key role in the cell-cell communication by functioning as endocrine and paracrine signals in diverse biological processes. Mode of action of exosomes secreted by immune cell types on immunological processes have been highlighted by several studies.

Exosome-mediated signaling is important for both benign and cancer cells. Secreted tumor-derived exosomes are part of the elements that compose liquid biopsy as a prospective novel tool in precision medicine [2]. In contrast with the limitations of tissue biopsy studies, the liquid biopsy offers possibilities of tumor identification reflecting in real time tumor heterogeneity, and are performed in a non-invasive way with biofluids, such as blood, urine, salvia or cerebrospinal fluid. Liquid biopsy enables the determination and identification of biomarkers useful for a variety of clinically relevant issues, such as cancer screening and early detection, real-time therapy monitoring, patient stratification, therapeutic interventions, risk of metastatic relapse, therapeutic target and resistance mechanism. Thus, liquid biopsy is currently the most innovative methodology in oncology shown to be effective in a number of tumor types. Elements of liquid biopsy studied include circulating tumor cells, circulating tumors nucleic acids free of cells or contained in exosomes, microvesicles and platelets [3]. Cell secreted EVs have gained attraction as they are physiological stabile and they contain molecules potentially to serve as biomarkers, such as lipids, glycans and metabolites [4]. In addition, mass spectrometric methods allow the study of protein profiles from blood borne nanoscale EVs to explore their potential for biomarker discoveries [5].

Several aspects make exosomes an ideal tool for translational cancer research, especially in patient-related fields with a high need for clinical improvement, so as to enable early cancer detection, avoid cancer progression and improve cancer therapy—see, e.g., [1,6]. The literature on exosomes and cancer strongly increased over the last decade with currently more than 10,000 articles published. Despite the recognized clinical potential, reliable analyses of exosomes remain methodologically challenging. More research on exosomes is needed to translate the existing knowledge from basic science and clinical studies into routine clinical applications. 

Exosome-based translational nanomedicine is a forward-looking approach using exosomes as biologically active carriers [1,7,8]. Exosomes can be used as a platform for enhanced delivery of cargo in vivo with potential for the development of clinical applications such as personalized therapies. However, a deeper knowledge of exosome biogenesis is required to overcome current limitations of producing sufficient amounts of safe and efficient exosomes. Understanding structural features and biosynthesis pathways of exosomes can improve production techniques important for clinical applications. It can be assumed that, e.g., novel reporter tools for observing EVs in live cells will support the basic research on EVs and advance the knowledge of both autocrine and paracrine roles of these nanostructures [9]. 

This Special Issue of Cancers covers translational research on exosomes in cancer from basic science to clinical studies, with a strong emphasis on improving the knowledge for clinical application. This call resulted in a total of 8 published articles, most being reports on original data and one review article. 

Hur and Lee supported this Special Issue with an overview review article that describes the current knowledge about DNA derived from EVs and its clinical applications [10]. This article highlights EV DNA and provides clues how EV DNA can be applied for diagnosis and prognosis as well as for therapeutic purpose. Moreover, not only double-strand DNA but also single-strand DNA has recently been detected in EVs consisting of specialized circular telomeric sequences suitable for diagnostic purposes [11,12]. 

The research articles of this Special Issue extend the knowledge about EVs for a broad range of cancer subtypes, such as glioblastoma [13], synovial sarcoma [14], pancreatic cancer [15], ovarian cancer [16], lung cancer [17], esophageal squamous cell carcinoma [18] and breast cancer [19]. 

The article from Godlewski et al. reports the influence of oncolytic virus infection on the secretome of human glioma stem cells with a focus on EVs [13]. This comprehensive study covers important topics that may be useful for the development of an oncolytic virus therapy for this devastating brain cancer subtype with limited therapeutic options. The described relations between EVs, oncolytic viruses and the immune responses contain important data for the glioma research field. 

Yokoo et al. describe a new liquid biopsy technique to sensitively monitor synovial sarcoma with a specific tumor marker on the surface membrane of circulating EVs [14]. Firstly, the marker monocarboxylate transporter 1 (MCT1) was identified in vitro by comparative proteome analyses and evaluated in a mice tumor model and human patients. In addition, the authors report that MCT1 may have therapeutic potential because its expression correlates with unfavorable patient outcome and a reduction in MCT1 expression in synovial sarcoma cells leads to decreased in vitro tumor characteristics such as cellular viability and migratory and invasive capacity. The presented data opens new possibilities for early diagnosis/prognosis and a more tailored therapy for this aggressive disease. 

Jung et al. described the composition of cytokine and growth factor profiles of serum and EVs isolated from breast cancer patients after neo-adjuvant chemotherapy and showed big differences [19]. The authors subsequently studied how the composition of the EVs or the serum correlated with clinic-pathological parameters of cancer and found that different molecules fulfill the criteria to function as a prognostic biomarker. In serum, high levels of IP-10 and MMP-1 indicate a shorter survival and in EVs, the presence of NGF after neo-adjuvant chemotherapy identifies breast cancer patients with bad survival prognosis [19]. 

Alharbi et al. determined the effects of hypoxia on the bioactivity of tumor-derived small EVs in ovarian cancer cells in vitro with focus on EV proteins [16]. The authors identified by a proteomic approach of several glycolytic pathway proteins that alter the metabolism of cancer cells, correlate with platinum resistance, and have the potential to be biomarkers of ovarian cancer recurrence in patients. As hypoxia is a general characteristic of tumors, these findings may not only be impactful in the field of ovarian cancer but also as well as for most cancer sub-types. 

Smolarz et al. compared lipid profiles of serum-derived small EVs from participants of a prospective lung cancer screening study with three groups of participants: without lung lesions, with benign lung nodules, and with screening-detected lung cancer [17]. Differences identified between groups were only of limited power with possible applicability as biomarker in the early detection of lung cancer. However, these descriptive findings extend the knowledge about the lipidome of serum-derived small EVs for early detection of lung cancer. 

Wu et al. investigated circulating exosome RNAs in pancreatic cancer [15]. A distinct exosome RNA gene signature was identified with high capacity of distinguishing pancreatic cancer from healthy individuals. The data indicate that circulating exosome RNAs have potential for early detection in pancreatic cancer. Such strategy for marker identification may also be applicable for other cancer subtypes in general. 

Song et al. isolated EVs from the plasma of esophageal cancer patients and showed that these vehicles included micro RNA mir-21-5p [18]. Additionally, they demonstrated that macrophage was able to take up EVs carrying mir-21-5p resulting in a disorganization of their polarization. In form of a feedback loop, the altered macrophages caused excessive migration and invasion of the esophageal cancer cells via the PTEN/AKT/STAT6 pathway. This paper presents a well-elaborated example of intercellular communication via exosomes in the background of esophageal cancer.

In conclusion, this Special Issue presents recent data covering translational research on exosomes in cancer from basic science to clinical studies. A wide range of molecules including RNA, DNA, proteins and lipids was shown to function as cues released by donor cells and able to induce cellular processes in recipient cells. It extends the knowledge about EVs over a broad range of cancer subtypes and provides a resource for clues how to continue this research field. The results described impact all aspects of cancer research, from prevention to more targeted therapy, and improve knowledge for clinical use. 
